# Near Vision Tasks and Optical Quality of the Eye

**DOI:** 10.18502/jovr.v16i4.9753

**Published:** 2021-10-25

**Authors:** Jessica Rafaela Moreira Gomes, Sandra Maria de Braga Franco

**Affiliations:** ^1^Centre of Physics, University of Minho, Campus de Gualtar, Braga, Portugal

**Keywords:** Computer, Near-vision Task, Optical Quality, Paper, Wavefront Aberrations

## Abstract

**Purpose:**

To study the effect of near-vision reading task on optical quality of the eye when performed on a computer monitor and on printed paper, and to identify which of the two results in greater changes.

**Methods:**

Two groups of subjects performed a 30-min reading task in two different conditions: on a computer monitor and on printed paper. Ocular, corneal, and internal wavefront aberrations (Zernike coefficients up to 6 ${}^{\mathrm{th}}$ order), root-mean-square of low- and high-order aberrations, spherical equivalent, vectoral components of ocular astigmatism (J45 and J0), and the compensation factor between internal and corneal aberrations were measured before and after the tasks. Their changes were analyzed in each group and between groups.

**Results:**

Statistically significant changes in wavefront aberrations and in root mean square of low- and high-order aberrations were observed in both groups which was significantly greater when the task was performed on printed paper. Partial loss of compensation mechanism and variation in spherical equivalent in a negative direction occurred after both reading tasks; however, it was statistically significant only with printed paper reading task. The vectoral components of ocular astigmatism did not show statistically significant changes in either groups.

**Conclusion:**

Near-vision reading tasks can change the optical quality of the eye, especially when the task is performed on printed paper.

##  INTRODUCTION

The use of near vision to perform different tasks is currently increasing, both at work and during entertainment. The use of digital devices such as computers, tablets, and smartphones to perform these tasks is also rising exponentially. Due to the excessive use of these devices, many studies have been conducted to address issues related to health and safety of the users.

Many ocular symptoms including eye strain, headache, ocular discomfort, double vision, and blurred vision at near and far distance are linked to the use of these devices.^[1,2]^ These symptoms are more common in subjects who spend over 4 hr on electronic devices and increase significantly in individuals who use electronic devices for more than 7 hr a day.^[2]^ Some studies were designed to explain the cause for these symptoms that affect around 90% of the electronic device users.^[1,3]^ Some studies^[1,2][3][4][5][6][7]^ reported the effect of computer screen use on visual system, and others^[7,10]^ about the same parameters but on the paper. In the study by Chu et al,^[8]^ the subjects performed two reading tasks on paper and on computer display at 50 cm distance for 20 min. The subjects then classified the intensity of the symptoms between 0 and 10. All symptoms were more intense during the reading task on computer except for blurred vision at far, which was worse for the task performed on paper. Therefore, it is important to understand if these symptoms are specific to the use of digital devices or they are manifestation of the increased use of near vision.

The accommodative response also changes with near vision tasks. There are studies about the changes in accommodative response after a near reading task on a digital device and on printed paper; however, the results are inconclusive. Penisten et al^[9]^ and Ferreira et al^[10]^ noted a higher accommodative response on computer reading task compared to paper. On the other hand, Wick and Morse^[4]^ measured the accommodative response in five emmetropic subjects reading a text on a computer screen and on hard copy. The accommodative lag showed more increase with the reading task on computer. Hue et al also found a higher increase in accommodative lag with computer use compared to printed copy.^[7]^ However, Collier and Rosenfield claimed that there were no changes in ocular accommodation of their 20 subjects after performing a reading task on computer for 30 min.^[5]^

Moulakaki et al concluded that the accommodative response appears to be independent of the type of electronic device used.^[11]^ They analyzed the accommodative response in 18 subjects for different accommodative demands (0, 1, 2, 3, and 4 D) after a 10-min reading task on two different electronic devices (tablet and smartphone) and did not find any statistically significant differences. On the other hand, Phamonvaechavan et al found a decrease in accommodation amplitude in 40 subjects after a 20-min continuous reading task which was more significant when the subjects used a tablet compared to when they used a computer screen.^[12]^ However, the focus of these studies was only on changes in ocular accommodation and the symptoms present in its use, and the results are still inconclusive.

Several studies found a transient myopia caused by near vision work, between –0.12 D and –1.30 D.^[13,14,15]^ Resolution of symptoms occurs 30/60 min after completing the task in symptomatic subjects.^[16,17]^

It has been suggested that the eyelid forces, during near vision tasks, causes significant changes in eye aberrations.^[18]^ Buehren et al noted that there was an increase in ocular high-order aberrations (HOA) following a 2-hr reading task which was more significant in myopic subjects and in eyes with large pupil diameters (4 and 5 mm).^[19]^

The changes in ocular aberrations depend also on the requirements of the tasks. The recent study of Jimánez et al found a higher increase in RMS astigmatism when subjects performed a task with higher-cognitive demands which persisted 10 min after completing the task.^[20]^

Previous studies showed that ocular aberrations change significantly with accommodation and the RMS of ocular low-order aberrations (LOA) and HOA increases.^[21,22,23,24,25,26,27]^ The most significant change is in the fourth-order spherical aberration, which is positive in the relaxed state and becomes negative with accommodation.^[24]^ The direction of the changes in coma and astigmatism is not clear.^[22,25]^ These changes in optical aberrations with accommodation can be explained by alterations in the shape and position of the lens during accommodation.^[21,28]^ Since most current daily tasks require ocular accommodation which in particular results in changes in eye aberrations, it is important to understand the effect of near-vision tasks on ocular, corneal, and internal optics.

It is already known that there is a partial compensation between cornea and internal optics.^[29,30,31,32,33]^ Artal et al found a compensation of the corneal coma and spherical aberration by the lens, leading to a better optical quality.^[29]^ A significant compensation of horizontal and vertical astigmatism, coma, and spherical aberration between cornea and lens were also observed in other studies.^[31,32]^ Some natural (such as age^[34,35]^) and artificial (such as ocular surgeries^[36,37,38]^) factors can alter this compensation effect and decrease the optical quality of the eye. As ocular accommodation during near-vision tasks changes the optical properties of the lens, the compensation mechanism might also be altered.^[21]^

There is evidence that near-vision tasks cause adverse symptoms and can lead to changes in spherical equivalent and accommodative response, and consequently might result in changes in wavefront aberrations and their balance between corneal and internal surfaces. However, it is not clear whether these changes are greater when the task is performed on paper or on digital devices. Studying the changes in optical quality of the eye with near-vision tasks can provide a better understanding of their effect on visual system. It can also help to understand which condition, paper or digital device, is more harmful. In addition, the origin of symptoms, which appears to be different on paper and electronic device reading tasks, can be explained by differences in optical quality.

The purpose of this study was to assess the effect of a near-vision task in different conditions (computer screen and paper) on ocular, corneal, and internal aberrations and on compensation mechanism. It was also intended to observe which condition affects these parameters more.

##  METHODS

### Subjects

The subjects were recruited from the students of the University of Minho. In a preliminary session, each one received a comprehensive optometric evaluation to ensure suitability for inclusion in the study. The examination included the assessment of visual acuity, objective and subjective refractive error, binocular vision, accommodation, and ocular health condition.

Two groups of subjects participated in the study; a group with 19 subjects (mean age 22.7 years; range, 19–25 years) who performed a reading task on a computer screen and another group with 34 subjects (mean age 20 years; range, 18–27 years) who performed the same task on printed paper. The sampling method was simple random.

All subjects in both groups were emmetropic with monocular uncorrected distance visual acuity of 20/20 or better. The emmetropic error was defined as a mean spherical equivalent ranging from –0.50 D to +0.50 D, and an astigmatism ≤ 0.50 D. The mean spherical equivalent of the subjects was +0.02 ± 0.27 D and +0.11 ± 0.25 D, and astigmatism –0.31 ± 0.15 D and 0.16 ± 0.18 D for computer and paper groups, respectively.

Moreover, subjects had no binocular and/or accommodative dysfunctions, history of ocular pathology or surgery, systemic disease, and they were not on any medication affecting vision. Normal binocular vision was defined according to Sheard criteria in cases with exophoria, and Percival criteria in subjects with esophoria.^[39]^

An informed consent was obtained from each subject after providing a verbal explanation of the nature and possible consequences of the study. The study was approved by the Ethics Subcommittee for the Life Sciences and Health of University of Minho.

### Experimental Procedure

The data collection encompassed two sessions: pre-task and post-task. For each subject, three measures of ocular and corneal wavefront aberrations of right eye were taken in each session and the mean was determined. After the first measurement, the subjects were seated comfortably and instructed to read a text at 50 cm distance (2.00 D of accommodative demand) in an illuminated room (photopic conditions) for 30 min. The text was a prose fiction in black letters and 12 font size, on a white background. In the computer group, the eye level was at the top of the computer screen, whereas in the paper group the printed text was placed on a table and therefore the subjects had downward gaze. All subjects were supervised to maintain the same position throughout the procedure.

Immediately after performing the reading task, the wavefront ocular aberrations were remeasured.

The acquisition was implemented under mesopic lighting conditions (150 lux) and natural pupil size (without mydriatic medication), ensuring a pupil diameter close to 5.0 mm during far and near distance measurements. For all procedures, ocular and corneal aberrations were then exported for a 5 mm pupil in the form of Zernike coefficients up to the sixth order.

The ocular and corneal aberrations were obtained before and after the task with Visionix VX 120 (Visionix Luneau, Chartes, France) analyzer, a closed view aberrometer. The repeatability of the measures intra-sections using this equipment was reported by a previous study^[40]^ and showed high levels for this parameter. The results of this study showed that all within-subject standard deviation (*S ${}_{w}$ *) for corneal power measurements were < 0.26 D, with Intraclass Correlation Coefficient (ICC) > 0.982. The *S ${}_{w}$* for corneal astigmatism at different areas (3, 5, and 7 mm) was < 0.21 D, with ICC > 0.913. Regarding the axis of astigmatism, its *S ${}_{w}$* was < 11.27º, with ICC > 0.975. The *S ${}_{w}$* and ICC for corneal eccentricity were 0.067 and 0.957, respectively. The *S ${}_{w}$* and ICC for HOA root mean square (RMS) were < 0.048 µm and > 0.901, respectively. third- and fourth-order aberrations, all *S ${}_{w}$* were < 0.037 µm and all ICC were > 0.84, except for quadrafoil RMS (ICC = 0.689). Moreover, according to Irene Sanchez et al, VX 120 device showed good reproducibility results, suggesting that this equipment is suitable for patients' follow-up, due to small differences between sessions.^[41]^

### Data Analysis

The values were exported in the form of Zernike coefficients up to sixth order, for 5 mm pupil diameter. The values were analyzed for this pupil diameter because it was the most approximate value of the natural pupil in our subjects. The first three Zernike terms were excluded from the analysis since they do not affect the image quality.

As the refraction based on wavefront aberration maps can accurately determine the sphero-cylindrical refraction,^[42]^ the spherical equivalent (M) was calculated using the paraxial curvature matching (i.e., the second-order paraxial focus, the fourth-order SA, and the sixth-order SA Zernike coefficients), using Equation (1):^[42]^


$$M=\left(-4\times \sqrt{3}\times Z_{2}^{0}+12\times \sqrt{5}\times Z_{4}^{0}-24\times \sqrt{7}\times Z_{6}^{0}\right)/r^{2},$$

where *r* is the radius of the pupil.

The two components of astigmatism, J45 and J0, were estimated by the least-squares fitting method, using Equations (2) and (3),^[42]^ respectively. 


$$J45=\left(-2\times \sqrt{6}\times Z_{2}^{-2}\right)/r^{2},$$

$$J0=\left(-2\times \sqrt{6}\times Z_{2}^{2}\right)/r^{2}.$$

The RMS of high-order ocular aberrations (HOA) were also calculated.

The contribution of the internal wavefront aberrations to the overall eye aberrations was investigated by subtracting corneal aberrations from the ocular aberrations.

Moreover, the compensation factor (CF) was calculated as:^[32]^


$$CF={(RMS}_{cornea}-{RMS}_{ocular})/{RMS}_{cornea}.$$

A positive CF indicates a compensation; values around zero, a lack of compensation; and negative values, addition of aberrations by the lens.

Data were analyzed using the IBM SPSS Statistics, Version 24.0. (IBM Corp, Armonk, NY; USA). The normality of the data was tested using the Shapiro–Wilk test. To compare the mean values between pre and post tasks in each study group, paired *t*-test was applied when the data followed a normal distribution; otherwise, Wilcoxon test was used. To evaluate the differences between groups, *t*-test and Mann–Whitney *U*-test were used for normal and non-normal distribution data, respectively. *P*-values ≤ 0.05 was considered statistically significant.

##  RESULTS

The ocular, corneal, and internal aberrations up to sixth order were measured before and after the reading tasks in both groups and their differences were analyzed and compared between the groups. The changes with the task for all Zernike coefficients from third to sixth order in both groups are shown in Table 1, and the statistically significant differences between the changes in both groups are marked.

In the computer group, no statistically significant differences were found in the ocular Zernike coefficients; however, corneal Z (5,–5) (*p* = 0.016), Z (5,–3) (*p* = 0.027), and Z (6,2) (*p* = 0.016) terms changed significantly. The same Zernike coefficients also showed statistically significant differences in the internal optics (*p* = 0.016, *p* = 0.031, and *p* = 0.012, respectively), as well as the term Z (6,4) (*p* = 0.049).

**Table 1:** Ocular, corneal, and internal Zernike coefficient mean changes [and standard deviations (SD)] of third, fourth, fifth, and sixth orders with the task, in the computer and paper groups, and the difference between their changes (computer change minus paper change)


	**Mean changes with the task (SD) (µ** * * **m)**						<**Difference between the changes of the computer and paper groups (µm)**		
	Computer			Paper					
	Ocular	Corneal	Internal	Ocular	Corneal	Internal	Ocular	Corneal	Internal
**Z (3,–3)**	0.006 (0.024)	–0.006 (0.068)	0.012 (0.063)	*0.026 (0.061)	–0.067 (0.229)	0.051 (0.240)	–0.020	0.061	–0.039
**Z (3,–1)**	–0.017 (0.053)	0.005 (0.005)	–0.021 (0.127)	*–0.021 (0.057)	*0.081 (0.227)	*–0.073 (0.226)	0.004	–0.077	0.051
**Z (3,1)**	–0.014 (0.043)	0.011 (0.085)	–0.025 (0.087)	–0.007 (0.032)	–0.002 (0.053)	*–0.080 (0.102)	–0.010	0.014	*0.055
**Z (3,3)**	–0.002 (0.026)	–0.044 (0.191)	0.042 (0.179)	–0.010 (0.039)	0.018 (0.125)	–0.025 (0.149)	0.007	–0.062	0.067
**Z (4,–4)**	–0.003 (0.019)	–0.034 (0.185)	0.031 (0.176)	–0.006 (0.029)	0.008 (0.105)	–0.012 (0.111)	0.003	–0.041	0.042
**Z (4,–2)**	0.0015 (0.015)	0.019 (0.086)	–0.017 (0.086)	0.002 (0.0197)	0.001 (0.052)	0.001 (0.052)	–0.001	0.018	–0.018
**Z (4,0)**	–0.001 (0.026)	–0.012 (0.040)	0.011 (0.049)	*–0.014 (0.030)	0.022 (0.101)	0.048 (0.100)	0.013	–0.034	*–0.038
**Z (4,2)**	–0.005 (0.022)	0.001 (0.036)	–0.012 (0.037)	*0.010 (0.041)	–0.032 (0.128)	*0.037 (0.127)	*–0.014	0.039	–0.049
**Z (4,4)**	0.001 (0.021)	0.003 (0.048)	–0.003 (0.060)	*–0.016 (0.037)	*0.037 (0.127)	–0.063 (0.120)	*0.016	–0.034	*0.060
**Z (5,–5)**	–0.004 (0.013)	*0.040 (0.077)	*–0.043 (0.079)	–0.001 (0.024)	–0.006 (0.088)	0.007 (0.083)	–0.003	0.046	–0.050
**Z (5,–3)**	0.002 (0.012)	*–0.017 (0.030)	*0.019 (0.035)	0.003 (0.026)	–0.007 (0.072)	0.009 (0.074)	–0.001	–0.010	0.010
**Z (5,–1)**	–0.006 (0.017)	0.000 (0.025)	–0.006 (0.028)	–0.008 (0.023)	0.011 (0.079)	–0.016 (0.077)	0.002	–0.010	0.010
**Z (5,1)**	0.001 (0.013)	0.007 (0.025)	–0.006 (0.027)	*0.001 (0.013)	0.001 (0.014)	–0.003 (0.022)	0.000	0.006	–0.003
**Z (5,3)**	–0.001 (0.011)	–0.011 (0.072)	0.010 (0.072)	0.005 (0.016)	–0.012 (0.042)	*0.016 (0.048)	*–0.006	0.001	–0.006
**Z (5,5)**	0.003 (0.011)	0.008 (0.110)	–0.005 (0.110)	–0.002 (0.023)	0.008 (0.086)	–0.013 (0.080)	0.005	0.000	0.008
**Z (6,–6)**	–0.001 (0.005)	0.006 (0.061)	–0.007 (0.064)	–0.003 (0.012)	0.004 (0.055)	–0.003 (0.057)	0.002	0.002	–0.003
**Z (6,–4)**	0.001 (0006)	–0.008 (0.040)	0.009 (0.040)	0.002 (0.012)	–0.004 (0.029)	0.002 (0.034)	0.000	–0.004	0.007
**Z (6,–2)**	–0.002 (0.005)	0.004 (0.027)	–0.004 (0.029)	–0.000 (0.007)	0.001 (0.019)	–0.001 (0.017)	0.000	0.003	–0.003
**Z (6,0)**	–0.001 (0.006)	0.002 (0.008)	–0.003 (0.009)	0.003 (0.014)	–0.001 (0.032)	0.002 (0.032)	–0.004	0.003	–0.005
**Z (6,2)**	0.002 (0.006)	*–0.007 (0.011)	*0.007 (0.011)	–0.001 (0.015)	0.001 (0.044)	–0.001 (0.039)	0.001	*–0.008	*0.009
**Z (6,4)**	–0.002 (0.007)	0.015 (0.041)	*–0.016 (0.037)	0.003 (0.013)	–0.004 (0.039)	0.011 (0.039)	–0.004	0.019	*–0.027
**Z (6,6)**	0.000 (0.007)	–0.018 (0.069)	0.018 (0.068)	–0.001 (0.012)	0.002 (0.062)	–0.010 (0.051)	0.001	–0.020	0.028
*Statistically significant difference								

On the other hand, the paper group had statistically significant differences in ocular, corneal, and internal aberrations. Cornea suffered significant differences in Z (3,–1) (*p* = 0.013) and Z (4,4) (*p* = 0.028) terms, internal optics in Z (3,–1) (*p* = 0.035), Z (3,1) (*p* < 0.001), Z (4,2) (*p* = 0.049), and Z (5,3) (*p* = 0.049) terms and ocular in Z (3,–3) (*p* = 0.019), Z (3,–1) (*p* = 0.027), Z (4,0) (*p* = 0.013), Z (4,2) (*p* = 0.018), Z (4,4) (*p* = 0.023) and Z (5,–1) (*p* = 0.046) terms.

The alterations caused by the task were compared between paper and computer groups [Table 1]. The changes observed after the task were greater in the paper group in several areas [ocular Z (4,2) (*p* = 0.004), Z (4,4) (*p* = 0.01), and Z (5,3) (*p* = 0.024); internal Z (3,1) (*p* = 0.025), Z (4,0) (*p* = 0.007), Z (4,4) (*p* = 0.033)], when compared to the changes in the computer group. On the other hand, corneal Z (6,2) (*p* = 0.014) and internal Z (6,2) (*p* = 0.006) and Z (6,4) (*p* = 0.001) showed greater changes in the computer group than in the paper group. Another Zernike terms showed no statistically significant differences between the changes observed with the task in the paper and computer groups.

It was also interesting to note that all corneal and internal Zernike coefficients of both groups, except Z (3,1) for the computer group and Z (3,1), Z (4,0), and Z (4,–2) for the paper group, changed in opposite directions. In other words, when the corneal aberration became more negative, the internal aberration became more positive, and vice versa. These opposite changes led to less alterations in ocular aberrations, despite some significant ocular and internal changes.

**Figure 1:**
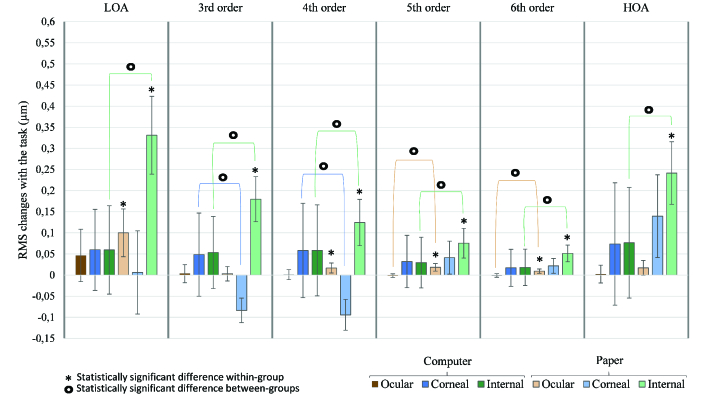
Changes in ocular, corneal, and internal RMS of third, fourth, fifth, and sixth-order aberrations and HOA between pre and post task conditions in both computer and paper groups, with 95%-confidence-interval error bars. There is a statistically significant increase of ocular, corneal, and internal RMS in several orders in the paper group and no statistically significant changes in the computer group. The changes in the paper group were significantly greater than the changes in the computer group.

The changes of ocular, corneal, and internal RMS of LOA, HOA, and third, fourth, fifth, and sixth orders were calculated in both groups and compared with each other [Figure 1].

In the computer group, the corneal and internal RMS of LOA and HOA, and the third, fourth, fifth, and sixth orders increased after the task; however, no statistically significant differences were observed.

On the other hand, in the paper group, ocular RMS of LOA (*p* = 0.001), fourth (*p* = 0.01), fifth (*p* < 0.001), and sixth (*p* < 0.001) orders had a statistically significant increase after the task. Moreover, corneal RMS of fifth (*p* = 0.01) and sixth (*p* = 0.025) order and all internal orders (*p* < 0.001 in all orders) changed significantly in this group.

The changes in RMS of ocular fifth (*p* = 0.001) and sixth (*p* = 0.007) orders, corneal third (*p* < 0.001) and fourth (*p* < 0.001) orders, and internal LOA (*p* < 0.001), HOA (*p* = 0.002), and third (*p* = 0.002), fourth (*p* = 0.006), fifth (*p* = 0.027), and sixth (*p* = 0.007) orders were significantly greater in the paper group than the changes found in the computer group. The values of the differences between the changes of these two groups are shown in Table 2, and the statistically significant differences are identified.

**Table 2:** Differences between the changes of the computer and paper groups in ocular, corneal, and internal low, third, fourth, fifth, and sixth, and high order root mean square


	**Difference between the changes of the computer and paper groups (µ** * * **m)**					
	**RMS LOA**	**RMS third order**	**RMS fourth order**	**RMS fifth order**	**RMS sixth order**	**RMS HOA**
**Ocular**	–0.054	0.000	–0.016	*–0.020	*–0.010	–0.015
**Corneal**	0.054	*0.132	*0.153	–0.009	–0.0048	–0.066
**Internal**	*–0.272	*–0.126	*–0.066	*–0.046	*–0.033	*–0.165
*Statistically significant difference						

**Figure 2:**
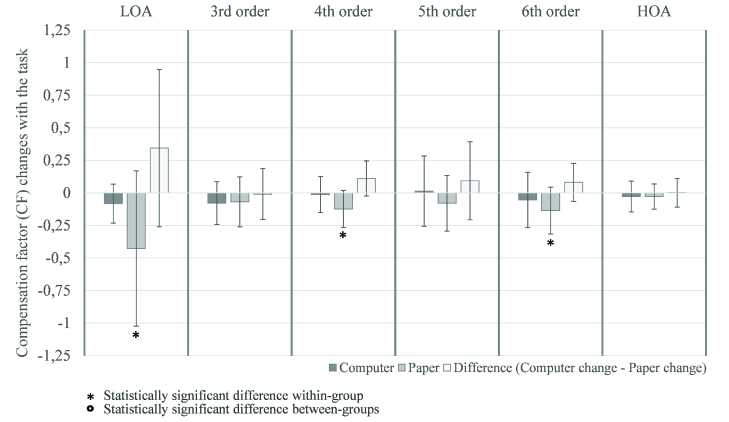
Compensation factor mean changes of LOA, HOA, and third, fourth, fifth, and sixth-order aberrations with the reading task in the computer and paper groups and the differences between their changes, with 95%- confidence-interval error bars. CF decreased with the task, and statistically significant changes were found in the paper group. There were no statistically significant differences between the changes found in the paper and computer groups with the task.

The CF was calculated to evaluate the compensation or addition of corneal aberrations by the internal optics before and after the task and to identify which task condition, computer or paper, cause more changes in this parameter. Their changes for LOA, HOA, and third, fourth, fifth, and sixth-order aberrations in both groups are represented in Figure 2. This parameter decreased in all orders after the task, except for the fifth order in the computer group, which has remained almost unchanged. However, the changes were only statistically significant in the LOA (*p* = 0.048) and fourth (*p* = 0.043) and sixth (*p* = 0.043) orders in the paper group.

Although the paper group showed more significant differences than the computer group, the differences between the two groups were not statistically significant, that is, none of the groups had variations in the CF significantly higher than the other.

**Figure 3:**
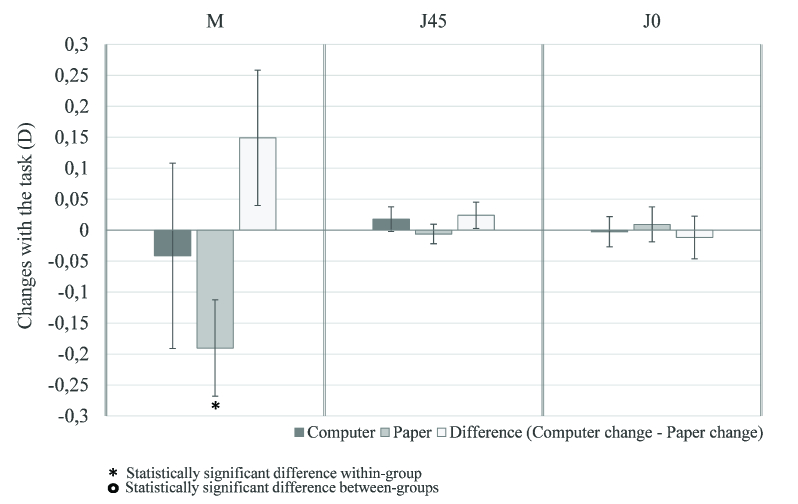
Changes of vector components of ocular astigmatism J45 and J0 and spherical equivalent of computer and paper group and the differences between their changes, with 95%-confidence-interval error bars. There were no statistically significant changes of astigmatism J45 and J0. Spherical equivalent became more negative with statistically significant change in the paper group. The changes found in both groups were not statistically significantly different.

The changes with the task of two vectoral components of ocular astigmatism (J45 and J0) were not statistically significant in either group [Figure 3]. Furthermore, none of the groups showed significantly greater differences than the other.

The spherical equivalent (M) was also calculated in pre and post task conditions and the mean differences were obtained in both groups [Figure 3]. The spherical equivalent became more negative after the task. Although, the changes were only statistically significant (*p* < 0.001) in the paper group, there were no statistically significant differences between the changes observed with the task in the paper and computer groups.

##  DISCUSSION

The focus of this study was to investigate the changes in eye optical quality after a near reading task in two different conditions, on a computer screen and on printed paper, and to compare them to each other.

For this purpose, the subjects in both groups read the same text for 30 min, and the ocular and corneal wavefront aberrations were measured before and after the task. Through Zernike coefficients, spherical equivalent, two vectoral components of astigmatism (J45 and J0), and RMS of HOA were calculated. Furthermore, the effect of near-vision tasks on compensation mechanism between corneal and internal optics was also investigated.

Changes in some corneal and internal Zernike coefficients were observed in the computer group [Table 1]. However, depending on how the corneal and internal aberrations changed in opposite directions, the increase of ocular aberrations was attenuated. Corneal and internal aberrations changed more significantly in the paper group, and caused variations in ocular aberrations, despite the reverse changes that had also occurred in this group.

The corneal wavefront variations found in this study are supported by the variations noted in previous studies,^[16,17]^ which showed changes in the corneal topography after reading, caused by the eyelid forces. However, as the position of the eyelids is different during the reading tasks on paper and on a computer screen, the differences of wavefront aberrations might be in different Zernike polynomials, depending on the position of the eyes during the task.

Furthermore, according to Ghosh et al the changes in ocular aberrations are greater when the subjects perform a task in downward gaze, (reading a paper) than when they perform a task in primary gaze (using a computer screen) which is in accordance with the results of this study.^[43]^

On the other hand, the variations in internal aberrations are attributed to the alterations resulted by ocular accommodation during near-vision task, which seems to maintain residual changes following the task.

The compensatory effect of wavefront aberrations between cornea and lens, already known in relaxed state,^[44]^ was also observed in this study; however, some changes were noticed following the task. The CF indicates that the task caused a general loss of compensation between corneal and internal aberrations which was statistically significant in the paper group [Figure 2].

Changes in the optical quality of eye components and loss of compensation can cause adverse symptoms in subject after performing a near-vision task. A previous study found more adverse symptoms with a task performed on a computer monitor compared to paper; however, blurred distance vision was more intensified with the task performed on printer paper.^[8]^ This finding is in accordance with the results of the present study that found more optical quality loss after the near-vision task on paper. A future study can also be conducted to evaluate and correlate the symptomatology during different vision tasks with the changes in optical quality. Furthermore, the decrease in pupil diameter induced by the luminance of the screen during the computer task (according to a previous study,^[45]^ there is a reduction of up to 20% in pupil size with tasks performed on electronic devices compared to printed paper) helps reduce high-order RMS and decrease blurred vision; contrary to the tasks performed on paper, during which the pupil diameter is larger, therefore resulting in a greater decrease in optical quality. Moreover, the reduction in pupil size caused by the luminance of the computer screen increases focus depth and thus decreases the accommodative effort necessary to maintain the text unblurred. Therefore, the changes in internal optics (caused by accommodation) are minor when the task is performed on a screen which does not occur on printed paper. The results of this study also support the above as the changes in internal optics were greater with paper compared to the computer.

The aberrations of the eye can be affected by several factors, such as accommodation^[46,47]^ and pupil diameter.^[48]^ According to Artal et al,^[49]^ this dynamic of ocular optics may compromise the efficiency of a refractive surgery that is only customized for static eye. This study also showed that near-vision tasks can change the corneal and internal optics of the eye and may affect the outcomes expected by refractive surgery.

Changes in compensation between corneal and internal aberrations were also reported by Qingzhong et al, who investigated this parameter during orthokeratology.^[50]^ In addition, they observed changes in internal and corneal aberrations in opposite direction for some Zernike polynomials [Z (3,–1), Z (3,1), Z (4,0), and Z (6,0)].

The changes in corneal wavefront aberrations caused by refractive surgery or orthokeratology may affect the compensation effect, not only in relaxed states, but also with near-vision tasks. Thus, these variations should be considered for the aberrometry performed to evaluate optical aberrations before and after the refractive surgery and orthokeratology.^[49]^ Furthermore, the compensatory effect between corneal and internal optics should be taken into account to improve vision quality after refractive surgery or orthokeratology.

The spherical equivalent became more negative after the reading task in this study, although this alteration was only statistically significant in the paper group [Figure 3]. These findings are consistent with previous studies^[13,14]^ and suggest that the transient myopia can occur when the task is performed on paper or on computer. Ghosh et al found changes in spherical equivalent in the negative direction during a task performed in downward gaze, even when there is no accommodation.^[43]^ As the paper task is performed in downward gaze, their finding is in agreement with the greater change in this group observed in our study. Several studies argue that this effect is involved in the development of myopia.^[15,19,51,52]^

There were no statistically significant differences in the vector components of astigmatism J45 and J0 in either group. However, analyzing the small changes that occurred, J0 became more negative with the task on computer (primary gaze) and more positive when the task was performed on paper (downward gaze). On the other hand, J45 became more positive in the computer group and more negative in the paper group. These findings were supported by the results of Ghosh et al's study^[43]^ that observed a similar trend even when the task was performed with no accommodation. Therefore, the eyelid forces, depending on their position during visual tasks, appear to be responsible for the changes in ocular astigmatism.

As previously mentioned, a study found highest increase in ocular astigmatism after tasks with higher cognitive demands.^[20]^ Future studies are required to analyze the changes in wavefront aberrations with more exigent tasks, to maintain a higher concentration of the subjects in the task, and the changes may be more significant. Moreover, it is important to understand the duration that these changes remain effective after the task is completed, and the impact on the eye of continuous.

In summary, near-vision tasks can affect ocular, corneal, and internal aberrations and ocular compensation. Consequently, these changes can cause adverse symptoms that are reported in this study. The changes are more significant when the subjects perform the task on a printed paper than when the task is performed on a computer screen.

##  Financial Support and Sponsorship

This work was supported by the Portuguese Foundation for Science and Technology (FCT) in the framework of the Strategic Funding UID/FIS/04650/2019 and by the project PTDC/FIS-OTI/31486/2017.

##  Conflicts of Interest

The authors have no conflicts of interest to declare.
